# Description of outcomes of experimental infection with feline haemoplasmas: Copy numbers, haematology, Coombs’ testing and blood glucose concentrations

**DOI:** 10.1016/j.vetmic.2009.06.028

**Published:** 2009-11-18

**Authors:** Séverine Tasker, Iain R. Peters, Kostas Papasouliotis, Simon M. Cue, Barbara Willi, Regina Hofmann-Lehmann, Timothy J. Gruffydd-Jones, Toby G. Knowles, Michael J. Day, Chris R. Helps

**Affiliations:** a School of Clinical Veterinary Science, University of Bristol, Langford, Bristol BS40 5DU, United Kingdom; b Clinical Laboratory, Vetsuisse Faculty, University of Zurich, Zurich, Switzerland

**Keywords:** Haemoplasma, Quantitative real-time PCR, Coombs’ test, Autoagglutination, Glucose

## Abstract

The aim of this study was to compare blood copy, haematological and glucose values between cats experimentally infected with either *Mycoplasma haemofelis* (Group HF: 10 cats), ‘*Candidatus* M. haemominutum’ (Group HM: 3 cats) or ‘*Candidatus* M. turicensis’ (Group TU: 3 cats). Blood samples were collected regularly up to 85 days post-infection (DPI) for haemoplasma real-time quantitative PCR, haematology, Coombs’ testing and blood glucose measurement. Statistical analysis was performed using a general linear model (ANOVA) appropriate for a repeated measures experiment with significance set as *P* < 0.05. Cats in Group TU had significantly lower blood copy numbers than cats in Group HF (*P* < 0.001) and HM (*P* < 0.001). All Group HF cats developed anaemia (often severe), macrocytosis and evidence of erythrocyte-bound antibodies whereas Groups HM and TU cats did not. Group HF had significantly lower PCVs, haemoglobin concentrations and red blood cell counts, and significantly higher mean cell volumes, than Groups HM and TU. In Group HF, erythrocyte-bound antibodies reactive at 4 °C (both IgM and IgG) appeared between 8 and 22 DPI and persisted for two to four weeks, whereas those reactive at 37 °C (primarily IgG) appeared between 22 and 29 DPI and persisted for one to five weeks. In most cats antibodies appeared after the fall in haemoglobin started. Although Group TU had significantly lower glucose concentrations than Groups HF (*P* = 0.006) and HM (*P* = 0.027), mean blood glucose concentrations remained within the reference range in all groups. This study demonstrates that *M. haemofelis* infection, in contrast to ‘*Candidatus* M. haemominutum’ and ‘*Candidatus* M. turicensis’ infection, can result in a severe macrocytic anaemia and the development of cold and warm reactive erythrocyte-bound antibodies.

## 1 Introduction

Three feline haemoplasma species are described; *Mycoplasma haemofelis*, ‘*Candidatus* M. haemominutum’ and ‘*Candidatus* M. turicensis’ (Foley and Pedersen, 2001; Neimark et al., 2001, 2002; Willi et al., 2005, 2006). *M. haemofelis* infection often causes haemolysis (Berent et al., 1998; Foley et al., 1998; Westfall et al., 2001) but ‘*Candidatus* M. haemominutum’ infection does not usually result in clinically significant anaemia (Foley et al., 1998; Westfall et al., 2001; Tasker et al., 2006a) without concurrent immunocompromisation (George et al., 2002). Experimental ‘*Candidatus* M. turicensis’ has only been evaluated in one previous study in which two cats developed anaemia secondary to infection (Willi et al., 2005) with more severe anaemia developing in the one cat that had been immunocompromised before inoculation.

Positive Coombs’ (direct antiglobulin) tests, indicating the presence of erythrocyte-bound antibodies, have been reported with haemoplasma infections in a number of species, including cats (Bundza et al., 1976; Harvey and Gaskin, 1978; Hoffmann et al., 1981). It has been proposed that such antibodies may contribute to the haemoplasma-associated haemolysis. However, investigations describing Coombs’ tests in haemoplasma-infected cats have varied greatly with respect to the methods described, or used, to perform Coombs’ testing (reagents, dilutions, temperatures employed and timing of sample collection) (Maede et al., 1974; Maede and Hata, 1975; Zulty and Kociba, 1990; Alleman et al., 1999; Willi et al., 2005). Additionally, studies have not yet described Coombs’ results in cats infected with each of the three feline haemoplasma species.

Severe hypoglycaemia has also been reported in several animal species acutely infected with haemoplasmas; sheep (*M. ovis*) (Sutton, 1977), an opossum (*Candidatus* M. haemodidelphidis) (Messick et al., 2000), pigs (*M. suis*) (Zachary and Smith, 1985; Smith et al., 1990), llamas (*Candidatus* M. haemolamae’) (McLaughlin et al., 1990; Messick et al., 2002) and calves (*Haemobartonella bovis*) (Love et al., 1977). Hypoglycaemia was reported in one moribund cat infected with a feline haemoplasma species (Harvey and Gaskin, 1977) but further details were not presented. A recent prevalence study found no significant difference in blood glucose concentrations between naturally haemoplasma infected and non-haemoplasma infected cats (Willi et al., 2006), although differentiation between acute and chronic haemoplasmosis was not possible.

The aim of this study was to describe and compare blood copy numbers and haematological changes, including Coombs’ test results, and blood glucose concentrations following experimental infection of cats with either *M. haemofelis*, ‘*Candidatus* M. haemominutum’ or ‘*Candidatus* M. turicensis’.

## 2 Materials and methods

### 2.1 Study design and protocol

Sixteen barrier-maintained specific pathogen (retroviral) free-derived domestic-shorthaired cats, aged 7 months, were used. Due to the variation in haemoplasma copy number anticipated with *M. haemofelis* infection (Tasker et al., 2003, 2006b), and the undertaking of a parallel study investigating subsequent haemoplasma copy number variation in the blood and tissues of some of the same cats (Tasker et al., in preparation), more cats were infected with *M. haemofelis* than the other two species. Ten cats were randomly assigned to the *M. haemofelis* group; Group HF (cats HF1 to HF4, HF6 to HF10 and HF12; six entire females and four neutered males), three to the ‘*Candidatus* M. haemominutum’ group; Group HM (cats HM1, HM2 and HM4; one entire female and two neutered males), and three to the ‘*Candidatus* M. turicensis’ group; Group TU (cats TU1, TU2 and TU4; three neutered males). The discontinuous numbering of the cats was due to the inclusion of additional cats in the parallel study (Tasker et al., in preparation) mentioned above.

Blood samples were collected on Day 0 of the study, immediately before haemoplasma inoculation. Blood was placed into EDTA-anticoagulant for complete blood count using a Cell Dyn 3700 analyser (Abbott, IL, USA), packed cell volume (PCV) determination (microhaematocrit tube centrifugation) and haemoplasma real-time quantitative PCR (qPCR), whilst a drop of remaining non-anticoagulated blood was used for blood glucose measurement (Accu-chek Aviva blood glucose system, Roche Diagnostics Ltd., Lewes, East Sussex, UK). Experimental inoculation of all cats with the respective haemoplasma species was carried out using heparinised blood collected from three barrier-maintained carrier donor cats. Cat blood types were predetermined (RapidVet-H blood typing cards, DMS Laboratories Inc., New Jersey, US) to be compatible (all type A). Following collection, the heparinised blood was placed on wet ice and 2 ml of heparinised donor blood was given intravenously to each of the 16 cats, via pre-placed cephalic intravenous catheters, within 5 min of collection. The haemoplasma inoculum doses comprised 1.55 × 10^8^ *M. haemofelis* copies, 5.04 × 10^6^ ‘*Candidatus* M. haemominutum’ copies or 2.42 × 10^6^ ‘*Candidatus* M. turicensis’ copies per cat.

Blood samples were collected from all cats three times weekly from 2 to 85 days post-infection (DPI): twice weekly, 0.3 ml blood was used for PCV, haemoplasma qPCR and blood glucose measurement, and once weekly, 1 ml blood was used for haematological examination, Coombs’ testing, PCV, haemoplasma qPCR and blood glucose measurement.

Cats were monitored daily for ill health (dehydration, pallor, anorexia) and were given subcutaneous fluid therapy (lactated Ringer's, up to 100 ml/kg/day) if they were pale, dehydrated or anorexic. Occasionally PCV was measured more frequently in individual cats if additional monitoring was needed to dictate therapy (data not shown). If PCV fell ≤10%, doxycycline at 10 mg/kg PO (followed by 3 ml water by syringe PO) was given daily until the PCV rose >10%.

All procedures and experiments described were undertaken under a project license approved under the UK Animals (Scientific Procedures) Act 1986.

### 2.2 DNA extraction

DNA was extracted from 100 μl EDTA-anticoagulated blood (Macherey-Nagel Nucleospin Blood kit, ABgene, Epsom, UK), eluting into 100 μl Buffer BE. For each batch of samples, blood samples from known haemoplasma non-infected and infected cats were subjected to DNA extraction as negative and positive controls respectively.

### 2.3 PCR assays

Haemoplasma qPCRs were carried out as previously described (Peters et al., 2008), using 5 μl of DNA template per 25 μl reaction. The assays consist of three distinct duplex qPCRs in which there is simultaneous detection of one of the haemoplasma species (*M. haemofelis*, ‘*Candidatus* M. haemominutum’ or ‘*Candidatus* M. turicensis’) and feline 28S rDNA as an internal control. DNA extraction and PCR analyses were performed on the days of blood collection. DNA samples from known haemoplasma-infected cats and water were subjected to qPCR as positive and negative controls respectively. Haemoplasma copy number quantification was derived by comparison to a standard curve generated by amplification of plasmids containing cloned 16S rDNA PCR products from each of the haemoplasma species (Tasker et al., 2003; Willi et al., 2005; Peters et al., 2008).

### 2.4 Coombs’ testing

Coombs’ testing was performed within 24 h of blood collection. An aliquot of EDTA-blood was suspended in an excess volume of cold phosphate buffered saline (PBS) (pH 7.4) and centrifuged (2200 × *g*, 3 min). The supernatant and buffy coat were removed before resuspending the pelleted red blood cells (RBCs) in PBS and repeating the centrifugation and removal of supernatant and buffy coat twice to wash the RBCs a total of three times. A 2.5% suspension of the packed, washed RBCs was then made in PBS. Serial dilutions (1:5–1:640) in PBS of polyvalent feline Coombs’ reagent (antiserum which reacts with feline IgG, IgM, IgA and C3; ICN Flow, Basingstoke, UK), anti-feline IgG Fc and anti-feline IgM Fc antiserum (both Nordic Laboratories, Tilburg, The Netherlands) were made (25 μl total) in two U-bottomed 96 well plates. Antisera were pre-absorbed with a pool of normal feline RBCs. Four control wells with PBS alone were also set up as negative controls. To each well, 25 μl of the 2.5% RBC suspension were added and mixed by tapping the plate. The two plates were then incubated for 1 h; one at 4 °C and one at 37 °C. The plates were examined for agglutination, with the recorded titre being the inverse of the reagent dilution in the last well where unequivocal agglutination occurred. Positive titres were described as being low if ≤80, moderate if 160 to 320 and high if ≥640. The titre, thermal activity and deduced antibody class of any positive cases were recorded. If agglutination was present in the PBS control wells, the titre and class of the antibody involved could not be recorded in the Coombs’ test and these cases were defined as having persistent autoagglutination, due to the presence of erythrocyte-bound antibody despite RBC washing, at the temperature at which it was demonstrated. Cats were described as having cold-reactive antibodies if persistent agglutination or Coombs’ positivity was only present or stronger at 4 °C compared to 37 °C, whereas those in which persistent agglutination or Coombs’ positivity was only present or stronger at 37 °C compared to 4 °C were said to have warm-reactive antibodies (Gehrs and Friedberg, 2002). Cats with equal persistent agglutination or Coombs’ positive reactions at both 4 °C and 37 °C were described as having both warm and cold-reactive antibodies (Gehrs and Friedberg, 2002).

### 2.5 Analysis of results

Data were explored by plotting haematological variables, blood glucose concentrations and haemoplasma copy numbers for each cat against time, and mean values of these variables for each group (HF, HM or TU) against time. The Coombs’ test results were tabulated, and summary Coombs’ results plotted against time alongside haemoglobin values to discern any pattern of Coombs’ reactivity in relation to onset of anaemia.

To evaluate for any effect of different haemoplasma species infection on haemoplasma copy number, haematological and blood glucose data, these variables were compared between each group (HF, HM or TU) using a general linear model (ANOVA) appropriate for a repeated measures experiment. The haemoplasma copy numbers were logged to allow a more biologically meaningful view of the variation in copy numbers recorded over time. Using these analyses, when a statistically significant difference was identified between the groups, the nature of the difference between individual groups was determined using the Tukey *post hoc* multiple comparisons test for normally distributed data. The residuals from this model were checked to ensure that the assumptions necessary for this parametric analysis were met.

When exploration of the data showed a fall in PCV in all three groups between Days 0 and 20 PI, to retrospectively evaluate whether haemoplasma infection with each of the species was associated with a significant change in PCV, a repeated measures ANOVA test was performed for all three groups from Day 0 to 20 PI. This test was applied retrospectively, after inspection of the data, and so the results can be viewed as a guide only.

Statistical analysis was performed using Statistical Package for Social Scientists (SPSS) version 14. Significance was taken at a *P* value of <0.05.

## 3 Results

### 3.1 Haemoplasma copy numbers

**Fig. 1:**
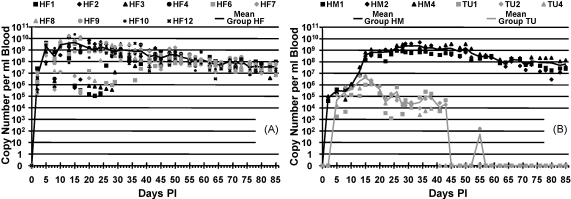
Haemoplasma copy numbers recorded during the study against time DPI. Copy numbers shown per millliter of blood. (A) *M. haemofelis* copy number/ml of blood for each cat in Group HF plotted against time DPI, with mean of Group HF plotted as continuous line. (B) ‘*Candidatus* M. haemominutum’ and ‘*Candidatus* M. turicensis’ copy number/ml of blood for each cat in Groups HM and TU plotted against time DPI, with mean of Groups HM and TU plotted as continuous lines. Group TU had significantly lower copy numbers than both Groups HF (*P* < 0.001) and HM (*P* < 0.001).

Fig. 1A and B shows the blood haemoplasma copy numbers recorded in Groups HF, HM and TU over time. All cats were haemoplasma PCR negative on Day 0 and became positive by 5 DPI. Some marked variation in *M. haemofelis* copy number was evident over time in each of the HF cats, especially until around 20 DPI, which was not evident in the other two infection groups other than mild variation in one of the TU group cats between 22 and 30 DPI. Mean maximum copy number was reached on 15 DPI in both the HF (∼4.4 × 10^9^ copies/ml blood) and TU groups (∼4.2 × 10^6^ copies/ml blood) but on 29 DPI in the HM group (∼2.5 × 10^9^ copies/ml blood). A repeated measures ANOVA showed that there was a highly significant (*P* < 0.001) day by group interaction on blood copy numbers. No significant difference (*P* = 0.936) in copy numbers between the HF and HM groups was found but the TU group had significantly lower copy numbers than both Groups HF (*P* < 0.001) and HM (*P* < 0.001).

### 3.2 Haematology

**Fig. 2:**
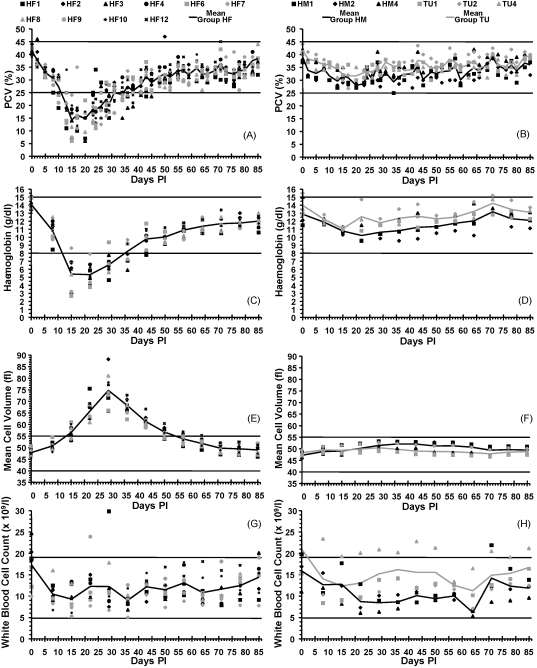
Selected haematological variables during the study against time DPI; horizontal lines represent upper and lower reference range limits for each variable. (A) PCV values for each cat in Group HF plotted against time DPI, with mean of Group HF plotted as continuous line. Reference range: 25–45%. (B) PCV values for each cat in Groups HM and TU plotted against time DPI, with mean of Groups HM and TU plotted as continuous lines. Reference range: 25–45%. Group HF had significantly lower PCVs than both Groups HM (*P* = 0.018) and TU (*P* = 0.001). (C) Haemoglobin values for each cat in Group HF plotted against time DPI, with mean of Group HF plotted as continuous line. Reference range: 8–15 g/dl. (D) Haemoglobin values for each cat in Groups HM and TU plotted against time DPI, with mean of Groups HM and TU plotted as continuous lines. Reference range: 8–15 g/dl. Group HF had significantly lower haemoglobin values than both Groups HM (*P* = 0.005) and TU (*P* < 0.001). (E) Mean cell volumes for each cat in Group HF plotted against time DPI, with mean of Group HF plotted as continuous line. Reference range: 40–55 fl. (F) Mean cell volumes for each cat in Groups HM and TU plotted against time DPI, with mean of Groups HM and TU plotted as continuous lines. Reference range: 40–55 fl. Group HF had significantly higher MCVs than both Groups HM (*P* < 0.001) and TU (*P* < 0.001). (G) White blood cell counts for each cat in Group HF plotted against time DPI, with mean of Group HF plotted as continuous line. Reference range: 4.9–19 × 10^9^/l. (H) White blood cell counts for each cat in Groups HM and TU plotted against time DPI, with mean of Groups HM and TU plotted as continuous lines. Reference range: 4.9–19 × 10^9^/l. White blood cell counts were not significantly different between the groups (*P* = 0.306).

Fig. 2 depicts haematological values recorded for all groups. All cats in Group HF group became anaemic (PCV <25%), being severe (PCV <15%) in 8 of the 10 cats with the mean lowest PCV value recorded on 15 DPI. None of the Group HM or TU cats became anaemic. A repeated measures ANOVA showed that there was a highly significant (*P* < 0.001) day by group interaction on PCV values. No significant difference (*P* = 0.319) in PCV was found between the HM and TU groups but Group HF had significantly lower PCVs than both Groups HM (*P* = 0.018) and TU (*P* = 0.001). Haemoglobin (Hb) concentrations and red blood cell counts (RBCC) displayed similar changes and statistical results to those seen with PCV. Mean cell volume (MCV) remained within the reference range in all Group HM and TU cats but rose above normal in all Group HF cats. A repeated measures ANOVA showed that there was a highly significant (*P* < 0.001) day by group interaction on MCV values. No significant difference (*P* = 0.614) in MCV between Groups HM and TU was found but Group HF had significantly higher MCVs than both Group HM (*P* < 0.001) and TU (*P* < 0.001). The white blood cell counts were not significantly different between the groups (*P* = 0.306).

A repeated measures ANOVA test performed in each group from 0 to 20 DPI found that all three haemoplasma species were associated with a significant PCV decrease over this time period: *P* < 0.001 for Groups HF and HM, and *P* = 0.001 for Group TU.

### 3.3 Coombs’ test

All cats were Coombs’ test negative on Day 0. Group HM and TU cats remained Coombs’ negative throughout the study whereas all cats in Group HF showed positive results at various time points. Those cats that showed persistent autoagglutination in the PBS control wells at 4 °C and/or 37 °C also showed agglutination in all test wells at that temperature, which precluded determination of the titre and class of the antibody involved at that temperature. Table 1 shows Coombs’ results from 0 to 64 DPI for all Group HF cats. Cold-reactive antibodies were seen at the majority of time points, whilst a moderate number of time points showed both cold and warm-reactive antibodies and a few showed warm-reactive antibodies.

**Fig. 3:**
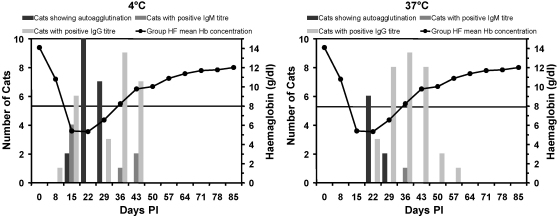
Graphs showing the collated autoagglutination and Coombs’ test data for all 10 Group HF cats, together with the Group HF mean haemoglobin values (reference range 8–15 g/dl) for temporal comparison to the time of onset of anaemia; horizontal lines represent upper and lower reference range limits. Note that if persistent autoagglutination was present, as shown by autoagglutination in the PBS control wells, the class of the antibody involved, at the temperature in which autoagglutination was demonstrated, could not be recorded. Hence cats with positive tests at either 4 °C or 37 °C will be graphically represented in either the autoagglutination bar chart or the IgG/IgM bar charts, but not both. (A) Number of cats in Group HF showing persistent autoagglutination, positive IgM titres or positive IgG titres at 4 °C alongside Group HF mean haemoglobin concentration. (B) Number of cats in Group HF showing persistent autoagglutination, positive IgM titres or positive IgG titres at 37 °C alongside Group HF mean haemoglobin concentration.

Regarding kinetics of the antibody response, erythrocyte-bound antibodies reactive at 4 °C appeared first, starting in all cats between 8 and 22 DPI and lasting for two to four weeks. Erythrocyte-bound antibodies reactive at 37 °C tended to appear slightly later, starting in all cats between 22 and 29 DPI and lasting for one to five weeks. Fig. 3 shows that in a few cats antibodies reactive at 4 °C appeared when the fall in Hb first became marked, whereas in the majority of cats they appeared at the nadir in Hb concentration. Antibodies reactive at 37 °C first appeared at the nadir in Hb concentration and were present in most cats as the Hb values increased again.

### 3.4 Blood glucose concentrations

**Fig. 4:**
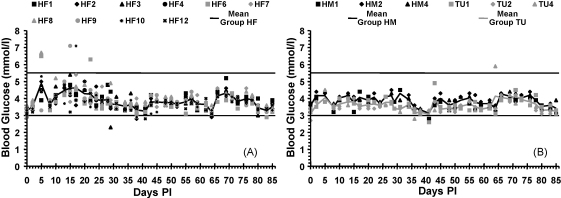
Blood glucose measurements during the study against time DPI; horizontal lines represent upper and lower reference range limits. Reference range: 3–5.5 mmol/l. (A) Blood glucose measurements for each cat in Group HF plotted against time DPI, with mean of Group HF plotted as continuous line. (B) Blood glucose measurements for each cat in Groups HM and TU plotted against time DPI, with mean of Groups HM and TU plotted as continuous lines. Group TU had significantly lower blood glucose concentrations than both Groups HF (*P* = 0.006) and HM (*P* = 0.027).

All three group mean glucose values remained within the reference range (Fig. 4), however a repeated measures ANOVA showed that there was a significant (*P* = 0.011) day by group interaction on blood glucose values. No significant difference (*P* = 0.997) in blood glucose concentrations between Groups HF and HM was found but Group TU had significantly lower blood glucose concentrations than both Groups HF (*P* = 0.006) and HM (*P* = 0.027).

### 3.5 Treatment

None of the Group HM or TU cats required treatment. Group HF cats were given subcutaneous fluid therapy due to dehydration, anorexia and/or pallor (HF1 on 8, 10, 12, 13, 14 and 15 DPI, HF2 on 13, 14, 15, 20 and 25 DPI, HF3 on 14 DPI, HF6 on 8, 10, 13, 14, 15 and 24 DPI, HF8 on 14 and 15 DPI, HF9 on 8, 10 and 13 DPI, HF9 on 8, 10 and 13 DPI, HF10 on 8 and 16 DPI, and HF12 on 10 and 13 DPI). Doxycycline was administered when PCV fell to ≤10% (HF1 on 14, 15, 17 and 20 DPI, HF2 on 14 DPI only, HF3 on 19, 20 and 22 DPI, HF6 on 15 and 24 DPI, HF8 on 20 DPI and HF9 on 15 DPI). HF6 was given doxycycline in error (PCV of 11%) on 24 DPI.

## 4 Discussion

This study is the first to describe contemporaneous experimental infection with either *M. haemofelis*, ‘*Candidatus* M. haemominutum’ or ‘*Candidatus* M. turicensis’ in cats of similar signalment maintained under identical conditions.

Group TU had significantly lower blood copy numbers than the HF and HM groups. The phenomenon of lower copy numbers with ‘*Candidatus* M. turicensis’ infection has previously been reported in naturally infected cats (Willi et al., 2006). Although the inoculum dose used for Group TU was far lower than for Group HF, it was only about half that used for Group HM, yet the maximal copy numbers reached in Groups TU and HM differed by several logs. Thus the lower TU group copy numbers are not likely due to inoculum dose alone. The HF and HM group copy numbers remained high for most of the study period whereas those of the TU group fell markedly after 38 DPI, and were negative in all TU cats by 45 DPI. These negative qPCR results may not have represented clearance of infection in all cats since TU2 and TU4 both generated positive qPCR results, albeit at very low copy numbers, later in the course of infection. However, these data suggest that spontaneous elimination of ‘*Candidatus* M. turicensis’ infection may be possible without treatment, as has been reported previously in two ‘*Candidatus* M. turicensis’ experimentally infected cats (Willi et al., 2005), although this was late in the course of infection in one cat (100 DPI).

The three feline haemoplasma species had differing pathogenicities. All 10 HF group cats had PCVs of <20% during the study, with seven and four cats reaching PCVs of <15% and <10% respectively. This is in contrast to a previous experimental *M. haemofelis* study using the same isolate (Tasker et al., 2006b) in which the induced anaemia was less severe. The inoculum dose used in the previous study (Tasker et al., 2006b) was not determined, although maximum *M. haemofelis* copy numbers in the cats of that and the current study were within one log of each other. Both studies comprised a mixture of neutered male and entire female cats, so gender differences are unlikely to be responsible. The cats in the current study were all 7 months of age, younger than the 5–8-year-old cats in the previous study (Tasker et al., 2006b). Age may play a part in the severity of *M. haemofelis*-induced anaemia, possibly due to immunological immaturity in younger animals (Day, 2007). Indeed, one study of naturally infected cats (Sykes et al., 2008) suggested that younger animals may be predisposed to clinical haemoplasmosis due to *M. haemofelis*.

Although ‘*Candidatus* M. haemominutum’ and ‘*Candidatus* M. turicensis’ were not associated with induction of anaemia or clinical signs, both were still associated with a significant drop in PCV up to 20 DPI, indicating an effect on RBC parameters, albeit not severe. Anaemia has been reported in two 10-year-old male neutered cats (Willi et al., 2005) following intravenous inoculation of the same isolate of ‘*Candidatus* M. turicensis’ that was used in the current study. However the fall in PCV was relatively mild in both cats with the more severe anaemia occurring in the cat given steroids prior to inoculation.

Group HF had significantly elevated MCVs, believed to reflect the presence of larger immature RBCs due to a regenerative response, although reticulocyte counts were not performed. A recent study reported that cats naturally infected with *M. haemofelis* had significantly elevated MCVs (Sykes et al., 2008), although concurrent feline leukaemia virus infection could have played a part in the development of macrocytosis in the cats of that study.

This study is the first to comprehensively describe Coombs’ testing during the course of experimental feline haemoplasmosis. Only HF cats showed positive results. Serum biochemistry was not performed routinely due to the need to restrict the blood volume collection but limited analysis (data not shown), performed between 22 and 36 DPI in the HF cats to monitor treatment, revealed hyperbilirubinaemia on 22 and/or 29 DPI in all but two Group HF cats. This hyperbilirubinaemia likely reflects haemolysis and was present when erythrocyte-bound antibodies were prominent.

All developed cold-reactive antibodies, consistent with the presence of IgM or IgG antibodies that fully or partially elute from the surface of the RBCs at physiological temperatures *in vitro*. Cold-reactive erythrocyte-bound antibodies have been reported previously with haemoplasma infection in cats (Zulty and Kociba, 1990), dogs (Bundza et al., 1976; Bellamy et al., 1978), and pigs (Hoffmann et al., 1981; Zachary and Smith, 1985), although the descriptions of the cold agglutinins and the method of detection used was inconsistent. Their significance is not known although some have suggested that they are involved in haemoplasma-induced anaemia (Zulty and Kociba, 1990). In this study the clear appearance and disappearance of such antibodies during *M. haemofelis*-associated anaemia suggests that infection induced the antibodies but it is difficult to assign a definitive pathophysiological role to the antibodies since the temperature at which the *in vitro* cold agglutination is observed does not reflect normal body temperature. However IgM is very efficient at complement fixation via the classical pathway, so it is possible that complement deposition onto RBCs occurs at the lower temperatures of the peripheral circulation (28–31 °C; Gehrs and Friedberg, 2002), even if the initiating antibodies then dissociate from RBCs at core body temperature. In humans, cold agglutinins may have thermal activity up to 30 °C (Rosse and Adams, 1980) and it is said that pathological cold agglutinins are characterised by having activity at such a temperature range (Gehrs and Friedberg, 2002). Although not reported here, we also evaluated the thermal reactivity of these antibodies by a further incubation at room temperature (18–20 °C), but the results of these tests mirrored those performed at 37 °C rather than 4 °C, suggesting that the cold agglutinins identified in our study did not have a wide thermal activity.

In three cats classical warm-reactive antibodies alone were identified at certain time points, but a larger number (eight) showed evidence of both warm and cold-reactive antibodies. Warm-reactive antibodies tended to appear after the cold-reactive antibodies, once the anaemia was established. Warm-reactive erythrocyte-bound antibodies have been previously demonstrated in haemoplasma-infected cats (Zulty and Kociba, 1990; Alleman et al., 1999) but descriptions of the nature of the warm antibodies and the method of detection used was inconsistent in those studies.

The role of warm-reactive antibodies in the pathogenesis of anaemia is less contentious, since these are active at normal body temperature, although they tended to appear after the anaemia had developed. Although cold-reactive antibodies appeared earlier in the course of infection, they were still not present at the time that Hb values started to fall. We cannot rule out that RBC bound antibodies were present before the onset of Coombs’ positive test results but that they were undetectable due to the sensitivity of the Coombs’ test being too low during early infection. Alternatively, it is possible that the erythrocyte antibodies arose merely as a sequel to *M. haemofelis* infection and/or haemolysis rather than initiating the anaemia. Binding of haemoplasma organisms to RBCs could unmask previously ‘cryptic’ RBC antigens and trigger the *de novo* induction of erythrocyte autoantibodies. The observed antibodies could also be haemoplasma-specific and be RBC-associated by virtue of the fact that the pathogen resides on the RBC surface. Previous studies have demonstrated that serum from haemoplasma-infected cats is able to agglutinate autologous parasitised RBCs and neuraminidase-treated non-parasitised RBCs but not normal RBCs (Zulty and Kociba, 1990), suggesting that cryptic RBC antigens may well be targeted by autoantibodies. Haemolysis itself may also expose such cryptic antigens.

Blood glucose concentrations in experimentally haemoplasma-infected cats have not previously been reported, other than a non-descriptive reference to a single case (Harvey and Gaskin, 1977), and two experimentally ‘*Candidatus* M. turicensis’-infected cats (Willi et al., 2005) in which no significant hypoglycaemia was seen although one cat, that had been immunosuppressed before inoculation, had glucose levels at the lower limit of the reference range but only starting after peak bacteraemia was recorded.^1^ Interestingly the current study found that Group TU cats had significantly lower blood glucose concentrations than the other two groups, although mean values remained within the reference range and no clinical effect was seen. Hypoglycaemia has been reported in other species (Love et al., 1977; Sutton, 1977; Zachary and Smith, 1985; McLaughlin et al., 1990; Smith et al., 1990; Messick et al., 2000, 2002), with the severity of hypoglycaemia being proportional to the degree of haemoplasma bacteraemia (McLaughlin et al., 1990). The reason for hypoglycaemia has not been fully elucidated although increased glucose utilisation has been demonstrated in *M. ovis*-infected RBCs (Sutton, 1976) and *M. suis* studies have suggested that this is due to the haemoplasma organisms themselves rather than the RBCs (Nonaka et al., 1996). Interestingly, many of the studies describing haemoplasma-associated hypoglycaemia fail to describe clinical signs consistent with significant hypoglycaemia (Zachary and Smith, 1985; McLaughlin et al., 1990; Smith et al., 1990; Messick et al., 2000), and two studies specifically describe marked hypoglycaemia in the absence of any associated clinical signs (Sutton, 1977; Burkhard and Garry, 2004). One of these studies (Burkhard and Garry, 2004) demonstrated the hypoglycaemia to be due to *in vitro* consumption by *M. ovis* since blood glucose measured immediately after collection from an infected lamb was normal, whereas the same blood submitted and ‘processed quickly’ (further details not given) revealed marked hypoglycaemia. The use of a handheld glucometer to measure blood glucose concentrations immediately in the current study may therefore have contributed to the observed normoglycaemia.

## 5 Conclusion

This study demonstrates lower ‘*Candidatus* M. turicensis’ blood copy numbers in cats infected with this species compared with the other two species. Only *M. haemofelis*-infected cats developed a macrocytic anaemia, and evidence of erythrocyte-bound antibodies which appeared after the onset of decreasing RBC parameters, suggesting that they did not directly mediate the anaemia. Hypoglycaemia was not found in this study.

## Figures and Tables

**Table 1:** Coombs’ test results for cats in Group HF.

Cat ID	Day PI	0		8		15		22		29		36		43		50		57		64	
	Coombs’ test	4 °C	37 °C	4 °C	37 °C	4 °C	37 °C	4 °C	37 °C	4 °C	37 °C	4 °C	37 °C	4 °C	37 °C	4 °C	37 °C	4 °C	37 °C	4 °C	37 °C
HF1	Polyclonal	0	0	160	0	Agg	0	Agg	20	Agg	80	0	0	0	0	0	0	0	0	0	0
	IgM	0	0	0	0	Agg	0	Agg	0	Agg	0	0	0	0	0	0	0	0	0	0	0
	IgG	0	0	80	0	Agg	0	Agg	160	Agg	320	160	80	0	0	0	0	0	0	0	0
	PBS	0	0	0	0	Agg	0	Agg	0	Agg	0	0	0	0	0	0	0	0	0	0	0

HF2	Polyclonal	0	0	0	0	80	0	Agg	Agg	Agg	Agg	20	20	0	20	0	0	0	0	0	0
	IgM	0	0	0	0	≥640	0	Agg	Agg	Agg	Agg	0	0	0	0	0	0	0	0	0	0
	IgG	0	0	0	0	320	0	Agg	Agg	Agg	Agg	320	160	160	80	0	0	0	0	0	0
	PBS	0	0	0	0	0	0	Agg	Agg	Agg	Agg	0	0	0	0	0	0	0	0	0	0

HF3	Polyclonal	0	0	0	0	40	0	Agg	Agg	160	40	40	20	20	20	0	20	0	0	0	0
	IgM	0	0	0	0	320	0	Agg	Agg	0	0	≥640	320	20	0	0	0	0	0	0	0
	IgG	0	0	0	0	160	0	Agg	Agg	≥640	≥640	320	160	320	160	0	≥640	0	160	0	0
	PBS	0	0	0	0	0	0	Agg	Agg	0	0	0	0	0	0	0	0	0	0	0	0

HF4	Polyclonal	0	0	0	0	40	0	Agg	Agg	80	40	20	20	0	0	0	0	0	0	0	0
	IgM	0	0	0	0	320	0	Agg	Agg	0	0	0	0	20	0	0	0	0	0	0	0
	IgG	0	0	0	0	80	0	Agg	Agg	≥640	320	320	160	320	320	0	0	0	0	0	0
	PBS	0	0	0	0	0	0	Agg	Agg	0	0	0	0	0	0	0	0	0	0	0	0

HF6	Polyclonal	0	0	0	0	Agg	0	Agg	Agg	320	20	0	0	0	20	0	0	0	0	0	0
	IgM	0	0	0	0	Agg	0	Agg	Agg	0	0	0	0	0	0	0	0	0	0	0	0
	IgG	0	0	0	0	Agg	0	Agg	Agg	≥640	160	160	80	≥640	320	0	0	0	0	0	0
	PBS	0	0	0	0	Agg	0	Agg	Agg	0	0	0	0	0	0	0	0	0	0	0	0

HF7	Polyclonal	0	0	0	0	0	0	Agg	0	Agg	40	20	20	40	40	0	0	0	0	0	0
	IgM	0	0	0	0	0	0	Agg	0	Agg	0	0	0	0	0	0	0	0	0	0	0
	IgG	0	0	0	0	0	0	Agg	0	Agg	≥640	≥640	≥640	≥640	≥640	0	0	0	0	0	0
	PBS	0	0	0	0	0	0	Agg	0	Agg	0	0	0	0	0	0	0	0	0	0	0

HF8	Polyclonal	0	0	0	0	20	0	Agg	Agg	Agg	80	0	20	40	40	0	20	0	0	0	0
	IgM	0	0	0	0	0	0	Agg	Agg	Agg	0	0	0	0	0	0	0	0	0	0	0
	IgG	0	0	0	0	320	0	Agg	Agg	Agg	≥640	≥640	≥640	≥640	≥640	0	≥640PZ80	0	0	0	0
	PBS	0	0	0	0	0	0	Agg	Agg	Agg	0	0	0	0	0	0	0	0	0	0	0

HF9	Polyclonal	0	0	0	0	80	0	Agg	0	Agg	20	0	0	0	0	0	0	0	0	0	0
	IgM	0	0	0	0	≥640	0	Agg	0	Agg	0	0	0	0	0	0	0	0	0	0	0
	IgG	0	0	0	0	80	0	Agg	80	Agg	320	0	0	0	0	0	0	0	0	0	0
	PBS	0	0	0	0	0	0	Agg	0	Agg	0	0	0	0	0	0	0	0	0	0	0

HF10	Polyclonal	0	0	0	0	0	0	Agg	30	Agg	Agg	20	0	0	0	0	0	0	0	0	0
	IgM	0	0	0	0	0	0	Agg	0	Agg	Agg	0	0	0	0	0	0	0	0	0	0
	IgG	0	0	0	0	0	0	Agg	320	Agg	Agg	160	160	160	160	0	0	0	0	0	0
	PBS	0	0	0	0	0	0	Agg	0	Agg	Agg	0	0	0	0	0	0	0	0	0	0

HF12	Polyclonal	0	0	0	0	20	0	Agg	Agg	Agg	80	0	0	0	20	0	0	0	0	0	0
	IgM	0	0	0	0	0	0	Agg	Agg	Agg	0	0	0	0	0	0	0	0	0	0	0
	IgG	0	0	0	0	80	0	Agg	Agg	Agg	≥640	≥640	320	0	≥640	0	0	0	0	0	0
	PBS	0	0	0	0	0	0	Agg	Agg	Agg	0	0	0	0	0	0	0	0	0	0	0

0 indicates negative. Numbers indicate the inverse of a positive titre to that reagent in the Coombs’ test. Agg indicates agglutination present in all wells. Results for Coombs’ testing done on Days 71, 78 and 85 PI are not shown as all cats were negative in all wells. PZ indicates prozone effect; this is observed when the concentration of antibody in the well is too high (i.e. at lower dilutions of Coombs’ antisera) to allow agglutination to occur. If the sample is diluted, agglutination then occurs. The lack of agglutination at high concentrations of antibodies is called the prozone effect. Lack of agglutination in the prozone is due to antibody excess resulting in very small complexes that do not clump to form visible agglutination.

## Footnotes

1 Personal communication BW, RHL.
